# A Rare Case of Triple-Negative Essential Thrombocythemia in a Young Postsplenectomy Patient: A Diagnostic Challenge

**DOI:** 10.1155/2018/9079462

**Published:** 2018-12-16

**Authors:** Tugce Akcan, Paolo Strati, Melissa Yan, Modupe Idowu

**Affiliations:** ^1^Department of Internal Medicine, University of Texas Health Science Center at Houston, Houston, TX, USA; ^2^Division of Cancer Medicine, University of Texas MD Anderson Cancer Center, Houston, TX, USA; ^3^Division of Hematology, University of Texas Health Science Center at Houston, Houston, TX, USA

## Abstract

The distinction between primary and reactive thrombocytosis by bone marrow histology is very important. Reactive thrombocytosis, the most common cause of thrombocytosis, can be expected in postsplenectomy states; however, close hematological evaluation of prolonged thrombocytosis is essential to identify patients who may have an underlying myeloproliferative neoplasm. We report a 37-year-old woman who was found to have portal, mesenteric, and splenic vein thrombosis with thrombocytosis, two months after she had a splenectomy for spontaneous splenic rupture. Other reactive conditions and myeloproliferative neoplasms (MPN) were excluded, and subsequently, the diagnosis of triple-negative essential thrombocythemia (ET) was established by bone marrow histology. This case of primary thrombocythemia following splenectomy in a young patient illustrates some of the diagnostic difficulties associated with postsplenectomy thrombocytosis. Continuing reports of anecdotal experiences in managing similar complex scenarios is essential and remains the only reference for clinicians facing these rare conditions.

## 1. Introduction

Essential thrombocythemia (ET) is a myeloproliferative neoplasm (MPN) and is characterized by increased number of mature megakaryocytes in the bone marrow and sustained thrombocytosis in the peripheral blood [1]. Difficulties may arise in differentiation from secondary thrombocytosis or other myeloproliferative diseases. We report an unusual presentation of triple-negative ET after splenectomy in a 37-year-old woman and demonstrate some of the problems associated with the diagnosis and management of the disorder.

## 2. Case Presentation

**Figure 1:**
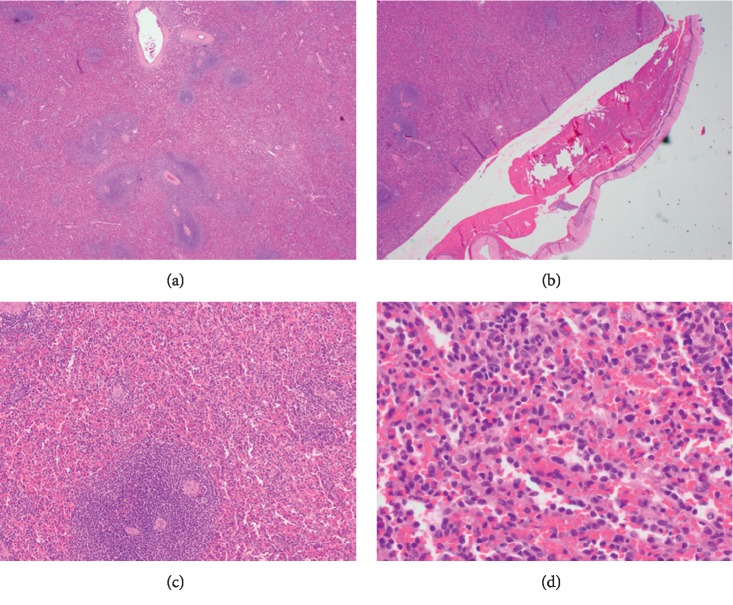
The histopathologic examination of spleen showed no abnormality except capsular rupture.

A previously well 37-year-old woman presented to the outside hospital following a near-syncopal episode. Initial workup included a computed tomography (CT) of the abdomen and pelvis, which revealed massive hemoperitoneum secondary to splenic injury. The patient denied any history of trauma, and no etiology of her splenic injury was discovered. She received an immediate urgent visceral angiography which included a selective angiography of the spleen. Angiography did not reveal any areas of active bleeding within the abdomen including from the spleen itself; therefore, embolization was not pursued. The patient was admitted to the intensive care unit where she underwent scrupulous monitoring. She continued to have tachycardia with an acute drop in her hemoglobin; hence, she was taken emergently to the operating room for exploratory laparotomy and splenectomy. The anatomopathological analysis of the spleen showed a 142 g ruptured spleen which measured 10.5 × 8.1 × 2.5 cm. On the anterior surface, was an area of rupture measuring 7.5 × 5.0 cm, which was surrounded by hemorrhage. The capsule was peeled back from this area. On the posterior aspect of the spleen, was an area of disruption measuring 5.0 × 2.2 × 0.1 cm, which was also surrounded by hemorrhage. The remaining capsule was lavender and smooth. The spleen was serially sectioned to reveal beefy red, smooth parenchyma with no gross lesions. Lymph nodes were not identified within the hilum. No gross lesions were identified. The histopathologic examination showed no abnormality except capsular rupture (Figure 1). She recovered well postoperatively and appeared improved at the time of discharge.

**Figure 2:**
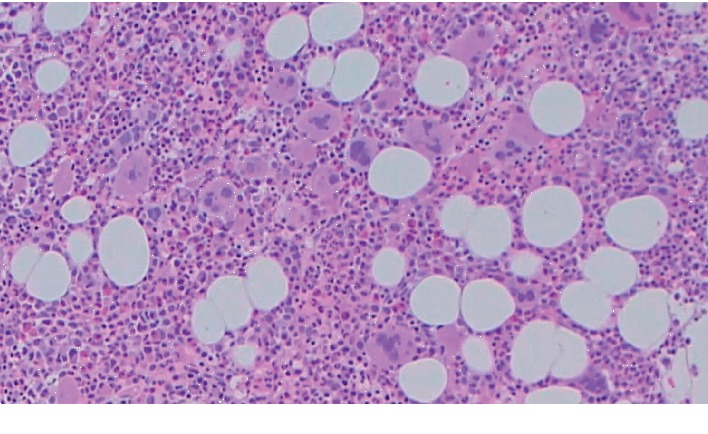
Bone marrow aspirate showing increased numbers of large megakaryocytes with hyperlobulation of megakaryocyte nuclei.

Two months later, the patient presented to our hospital with complaints of acute, severe, diffuse abdominal and low back pain. She had tachycardia while the other vital signs were normal. Laboratory studies revealed a hemoglobin of 10.5 g/dL, hematocrit of 33.9%, neutrophilia of 13.2 × 10^9^/L, and thrombocytosis of 2239 × 10^9^/L. CT abdomen with contrast revealed portal, mesenteric, and splenic vein thromboses. She denied any smoking history, had no family history of thrombophilia, and was not taking oral contraceptives. Thrombophilia workup (Factor V Leiden mutation and prothrombin gene mutations, protein C, protein S, antithrombin III, homocysteine levels, and antiphospholipid antibodies) was unremarkable. The erythropoietin level was high at 30 mIU/ml (normal range 2.6 to 18.5 mIU/ml). Other causes of reactive thrombocytosis, including infection, inflammation, trauma, and acute blood loss, were not identified. Bone marrow biopsy revealed hypercellularity (60% for age) with a marked increase in enlarged and mature hyperlobulated megakaryocytes, compatible with essential thrombocythemia (ET) (Figure 2). There was no abnormality seen in granulocytic or erythroid precursors; both showed adequate number with normal maturation. Iron stores were adequate, and there was no evidence of fibrosis.

Another independent pathologist reviewed the bone marrow biopsy and confirmed the initial findings. The patient's cytogenetic profile showed normal female karyotype, 46, XX. BCR-ABL, JAK2 V617F, CALR (not specified to type 1 or 2), and MPL W515L/K gene mutations were negative. The patient was diagnosed with essential thrombocythemia [1].

The patient received one urgent therapeutic platelet apheresis without complications, which resulted in the temporary removal of the majority of platelets [2]. Low-dose aspirin, low molecular weight heparin, and hydroxyurea 1000 mg daily were initiated. However, her platelet count continued to increase despite increasing hydroxyurea dose to 1500 mg daily; therefore, anagrelide 0.5 mg PO BID was added. At the time of discharge, the patient was asymptomatic and platelet count had decreased to 664 × 10^9^/L. Due to intolerance, the anagrelide was discontinued on day 75. Six months later, hydroxyurea dose was decreased to 1000 mg daily, and she remains on this dose. At the time of this report, the patient has an ongoing positive response to treatment and platelet count has further decreased to 445 × 10^9^/L (Table 1).

## 3. Discussion

Essential thrombocythemia (ET) is an acquired myeloproliferative neoplasm (MPN) characterized by clonal proliferation of megakaryocytes and thrombocytosis [1, 3]. The other common types of thrombocytosis include reactive (or secondary) thrombocytosis, clonal myeloid neoplasms, and familial or hereditary thrombocytosis. Reactive thrombocytosis is the most common cause of thrombocytosis and can be secondary to infections, iron deficiency anemia, postsurgical status, malignancy, major trauma, and/or postsplenectomy state [4]. ET is a diagnosis of exclusion and requires the absence of reactive conditions and other clonal disorders.

The distinction between primary thrombocythemia and reactive thrombocytosis may be difficult. In postsplenectomy reactive thrombocytosis, accounting for 9% of all reactive thrombocytosis, the platelet count peaks at 1 to 3 weeks and subsides within weeks to months [5]. Portal, mesenteric, and splenic vein thrombosis in association with the elevated platelet count has been recognized, with an incidence of 5% postsplenectomy, and is identified within two weeks of the operation [6]. However, studies suggest that thrombosis occurs most often when thrombocytosis is due to an underlying hematological neoplasm rather than a reactive condition, and thrombocytosis status after splenectomy has not been associated with an increased risk for thrombosis [7].

The patient described in this report was atypical because she had a normal platelet count prior to splenectomy, and ET was unmasked by a splenectomy performed for other reasons. To our knowledge, this is the first case reported in the literature of splenectomy unmasking ET with the thrombotic complications. This case demonstrates the diagnostic difficulty with primary thrombocythemia, especially in a young patient. However, the degree and duration of the thrombocytosis, presenting with a thrombotic event combined with the very characteristic morphological appearances in the bone marrow, virtually favor neoplastic over reactive thrombocytosis in our patient.

From the genetic perspective, ET patients harbor mutations in *JAK2*V617F (50–60%), *CALR* (15–30%), and *MPL*W515L/K (1–5%) genes. After excluding mutations in these three genes, a proportion of ET patients still do not harbor any identifiable mutation. This subgroup of patients is categorized as “triple-negative” (TN) [8]. Part of the variability in clinical outcomes of patients with ET is likely related to the founding or initiating mutation, and genomic profiling holds the potential to improve prognostication and clinical decision-making. Studies have shown that *JAK2*-positive patients have a shorter thrombosis-free survival and the presence of a *JAK2* V617F mutation retained its strong negative prognostic relevance in terms of predicting thrombosis [9]. Triple-negative ET patients seem to have remarkably favorable overall survival and had few cardiovascular events during the follow-up period. In addition, triple-negative ET patients usually present at a younger age, have lower hemoglobin level and leukocyte count, and have a lower incidence of thrombosis compared with mutated cases. In contrast, triple-negative primary myelofibrosis patients have been associated with a worse overall survival and a higher incidence of leukemia. Leukemia-free survival in ET was not affected by mutational status [10].

ET is most commonly complicated by vascular events, including both thrombosis and bleeding. Arterial and venous thromboses, as well as platelet-mediated transient occlusions of the microcirculation and bleeding, represent the major cause of mortality associated with ET [11]. The major risk factors for thrombosis are previous thrombotic episode and age older than 60 years, whereas recent data suggest a prognostic role for novel risk factors, including leukocytosis and mutational status [9, 10, 12]. Although the patient's thrombosis risk was low prior to her diagnosis of splanchnic vein thrombosis, it is possible that the splenic rupture and previous abdominal surgery were additional risk factors for thrombosis. She may also have had a small thrombosis close to the time of splenic rupture and previous abdominal surgery, which may have progressed with the onset of severe thrombocytosis. The patient had a very thorough workup for malignancy and liver disease, which were unremarkable. Any other underlying pathology was unable to be identified.

Atraumatic spontaneous splenic rupture is rarely documented to be associated with myeloproliferative neoplasms especially with polycythemia vera (PV). To date, there are only five cases in world literature of atraumatic spontaneous splenic rupture associated with PV [13–17]. All five cases presented with splenomegaly and hemodynamic instability and required an exploratory laparotomy at which time a splenectomy was performed. Besides, PV has already been diagnosed or the presence of PV had been suspected before splenic rupture. Suboptimal treatment and noncompliance with medications or follow-up appointments are common between the cases diagnosed with PV. Suggested underlying mechanisms include those various pathologic entities of PV that lead to hypercoagulable state and increase the risk for splenic infarction, which may lead to subsequent rupture and hemorrhage. WHO diagnostic criteria require the presence of either all three major criteria (elevated hemoglobin, myeloid hypercellularity, and JAK2 V617F mutation) or the first two major criteria and the minor criterion (subnormal serum erythropoietin) [1]. Seventy-five to ninety percent of patients with PV have the triad of splenomegaly, polycythemia, and ruddy cyanosis. Retrospectively, our patient was healthy and complete blood count (CBC) was normal before splenectomy was performed. At that time, spleen size and the pathology report of the spleen were normal. No increase in granulopoiesis or erythropoiesis was observed in bone marrow biopsy specimen. Iron stores were adequate, and there was no evidence of fibrosis. JAK2 V617F gene mutation was tested and negative. The serum erythropoietin level was elevated. Considering our patient's presentation, diagnostic results, and the factors mentioned above, we think that PV is unlikely the underlying cause of atraumatic spontaneous splenic rupture and splenectomy of our patient.

Prevention of vascular events has been the main objective of therapy and continues to be extremely important in the management of patients with ET [18]. Low-dose aspirin and cytoreductive drugs can be administered for this purpose, and cytoreductive therapy with hydroxyurea [19], interferon alpha [20], or anagrelide [21] has been effective in preventing thrombotic episodes in high-risk patients.

This rare case of primary thrombocythemia in a young patient with a history of splenectomy illustrates some of the diagnostic difficulties in this disorder. Thrombocytosis is an expected postsplenectomy finding; however, close hematological monitoring and a high index of suspicion of a persistent thrombocytosis is necessary to identify rare patients with an underlying myeloproliferative disorder.

## Figures and Tables

**Table 1:** Trend of patient's complete blood count (CBC) over the hospital stay with respect to timing of admission.

	Plt (10^9^/L)	WBC (10^3^/*μ*L)	RBC (10^6^/*μ*L)	Hgb (g/dL)	Hct (%)
Splenectomy (2 months prior to admission)	140	24.2	2.73	7.8	22.8
Splanchnic venous thrombosis (admission)	2239	24.6	3.95	10.4	33.4
Beginning of plateletpheresis session (day 2)	2079	21.6	3.96	10.3	33.2
End of plateletpheresis session (day 2)	402	16.4	4.32	11.1	36
Started hydroxyurea 1000 mg PO daily (day 4)	670	13.8	3.17	8.5	26.2
Increased dose of hydroxyurea 1500 mg PO daily (day 8)	1307	11.5	3.31	9	27.7
Started anagrelide 0.5 mg PO BID (day 10)	1595	14.4	3.63	9.9	30.9
Discharged home (day 19)	644	11.2	3.63	10.1	31.2
Anagrelide 0.5 mg PO BID discontinued (day 75)	397	8.5	3.1	11.3	33.9
Decreased hydroxyurea 1000 mg PO daily (6 months)	512	8.8	3.12	12.5	36.8
